# Hydraulic fracturing analysis in fluid‐saturated porous medium

**DOI:** 10.1002/nag.3447

**Published:** 2022-09-11

**Authors:** Lin Chen, Farshid Fathi, Rene de Borst

**Affiliations:** ^1^ Key Laboratory of Ministry of Education on Safe Mining of Deep Metal Mines Northeastern University Shenyang China; ^2^ Department of Civil and Structural Engineering University of Sheffield Sheffield UK

**Keywords:** Powell‐Sabin B‐splines, hydraulic fracturing, cohesive zone model, porous medium, remeshing

## Abstract

This paper addresses fluid‐driven crack propagation in a porous medium. Cohesive interface elements are employed to model the behaviour of the crack. To simulate hydraulic fracturing, a fluid pressure degree of freedom is introduced inside the crack, separate from the fluid degrees of freedom in the bulk. Powell‐Sabin B‐splines, which are based on triangles, are employed to describe the geometry of the domain and to interpolate the field variables: displacements and interstitial fluid pressure. Due to their $C^{1}$‐continuity, the stress and pressure gradient are smooth throughout the whole domain, enabling a direct assessment of the fracture criterion at the crack tip and ensuring local mass conservation. Due to the use of triangles, crack insertion and remeshing are straightforward and can be done directly in the physical domain. During remeshing a mapping of the state vector (displacement and interstitial fluid pressure) is required. For this, a new methodology is exploited based on a least‐square fit with the energy balance and mass conservation as constraints. The accuracy to model free crack propagation is demonstrated by two numerical examples, including crack propagation in a plate with two notches.

## 1 INTRODUCTION

Production of natural gas and oil from hydrocarbon‐rich shale formations has become popular and exploits hydraulic fracturing at a large scale. Yet, hydraulic fracturing as a well stimulation technique for improving hydrocarbon production has been known since the late 1940s. The technique involves the fracturing of a porous medium by a pressurised liquid. The process consists of high‐pressure injection of a fracking fluid into a well‐bore to create cracks in the deep porous medium. In the early stages of hydraulic fracturing modelling, analytical solutions^1^, ^2^ were derived on the basis of simplifying assumptions, such as homogeneity and impermeability, an idealised geometry and linear elasticity. The first numerical model of the fluid flow in a porous medium with a discontinuity was done by Boone and Ingraffea,^3^ using finite elements for the porous medium and finite differences for the fluid in the crack. Since then a host of numerical models have been proposed, such as finite elements,^4^ the extended finite element method,^5^, ^6^, ^7^ isogeometric analysis,^8^ extended isogeometric analysis,^9^ embedded strong discontinuities,^10^ the phase‐field method,^11^ a coupled finite element‐peridynamics model,^12^ interfaces elements equipped with a cohesive zone model^13^ and a combined finite‐discrete element method.^14^

Due to their simplicity and robust performance interface elements have gained popularity for modelling fracture initiation and propagation in a poroelastic medium.^15^, ^16^ The interface elements are placed in the mesh a priori, requiring information of the crack location. This limits the application of the interface elements in a general framework. Remeshing can however remove this limitation and has been used successfully in the simulation of fracturing in a porous medium.^17^, ^18^

For the interpolation of the field variables several basis functions have been employed in this context. Lagrange basis functions have been used frequently,^15^, ^16^ due to their simplicity and ease of implementation. However, the $C^{0}$‐continuity nature of the basis function deteriorates the accuracy of the simulation. The stress is generally discontinuous at element boundaries and the crack tip. Normally, several elements are needed to well capture the fracturing behaviour, such as the crack initiation and propagation direction. Furthermore, $C^{0}$‐continuous Lagrange bases lead to a discontinuous inter‐element pressure gradient. Accordingly, a local mass balance is not be guaranteed.

Due to their higher‐order continuity, the basis functions used in isogemetric analysis – NURBS and T‐splines – normally avoid the discontinuous stress field and a loss of local mass balance, and have been used for discrete crack modelling,^19^ including in simulations of fracture in a fluid‐saturated medium.^8^, ^9^ However, in discrete crack modelling the isogeometric approach requires the introduction of $C^{0}$‐lines in cracked elements to confine the influence of cracks locally,^20^ thus eliminating locally the advantage of isogeometric analysis, namely higher‐order continuity. Moreover, new crack segments are inserted in the parameter domain, rather than in the physical domain, and a reparameterisation of the domain is required to align the mesh with the crack path in the physical domain. This makes it mandatory that the initial mesh is sufficiently aligned with the final crack path a priori.^20^

In this contribution, we employ Powell‐Sabin B‐splines to simulate hydraulic fracturing in a fluid‐saturated porous medium. Powell‐Sabin B‐splines are defined on triangles, holding $C^{1}$‐continuity throughout the entire domain, even at crack tips. This avoids the inaccuracy of the stress evaluation when employing Lagrange basis functions. Due to the flexibility of triangular elements crack insertion is carried out directly in the physical domain, thus avoiding the limitation adhering to isogeometric analysis. After the crack insertion the domain is remeshed to avoid elements with unsuitable aspect ratio, resulting in new Powell‐Sabin B‐splines on a new mesh. Then, a mapping of the state vector (displacements and pressure) is performed from the old onto the new mesh.

We start with an introduction of the governing equations for the hydraulic fracturing analysis. Section  3 derives the weak form of the governing equations. Next, we present the Powell‐Sabin finite element discretisation. The basis functions and poromechanical interface elements are introduced here. In Section 5, we discuss the algorithm to insert a new crack segment, including the algorithm for remeshing and state vector mapping. In Section 6, numerical examples are given which demonstrate the versatility and accuracy of the method.

## 2 GOVERNING EQUATIONS FOR THE POROUS MEDIUM

**FIGURE 1:**
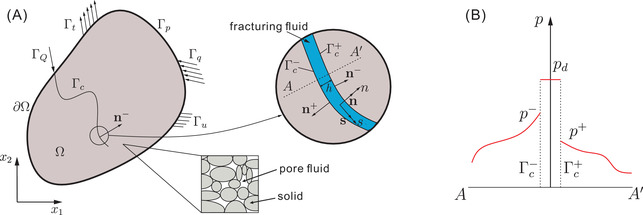
(A) A solid body Ω with an internal discontinuity $\Gamma_{c}$. $\Gamma_{c}$ is an interface boundary with positive and negative sides, $\Gamma_{c}^{+}$ and $\Gamma_{c}^{-}$, respectively. Boundary $\Gamma_{u}$ is prescribed with a displacement $\bar{u}$; $\Gamma_{t}$ with a prescribed traction $\hat{t}$; $\Gamma_{p}$ with a prescribed fluid pressure $\bar{p}$; $\Gamma_{q}$ with a prescribed inflow $\hat{q}$; and $\Gamma_{Q}$ with a prescribed inflow of fluid $\hat{Q}$; (B) pressure around the internal discontinuity $\Gamma_{c}$.

Hydraulic fracturing in porous media is a complex physical phenomenon, including fluid flow in fractures, the pore fluid flow in the porous medium, and the deformation of the porous medium. In this contribution, we confine the discussion to (1) a fully saturated porous medium, (2) a Newtonian fluid, (3) infinitesimal strains and linear elastic material behaviour, (4) no mass transfer or chemical interaction between the solid and the fluid, (5) no consideration of gravity, and convective and inertia effects and (6) the fluid fully occupying the fracture, thus not considering possible fluid lag. We refer to Figure 1 for a graphical illustration of the investigated problem. The porous medium is split into two parts by an interface $\Gamma_{c}$. Biot's theory is used to model the porous media.^21^ The cohesive‐zone model is used to model the fracturing behaviour.^22^, ^23^

### 2.1 Deformations of the porous media

The fully saturated porous medium is modelled as a two‐phase system with the solid skeleton fully filled with pore fluid. The deformation of the solid develops fast compared to the pore fluid pressure change. Thus, the deformation of the porous media can be considered as a quasi‐static process, governed by the conservation of the hydro‐static linear momentum^24^:

(1) ∇·σ=0onΩ

where σ is the total stress, composed of the solid and pore fluid parts

(2) $$\sigma =\sigma_{s}-\alpha pI$$

in which *p* represents the apparent fluid pressure, I denotes the unit tensor and α is the Biot coefficient. $\sigma_{s}$ is the stress inside the solid material, which is linearly related to the strain by

(3) $$\sigma_{s}=D:\varepsilon$$

with **D** the elastic stiffness tensor, which is a function of two material constants – the Young's modulus and the Poisson's ratio for isotropy.

### 2.2 Interstitial fluid pressure

The governing equations of the pressure in the interstitial fluid are obtained from the mass balance of the mixture^13^

(4) $$\alpha \nabla \cdot \dot{u}-\nabla \cdot \left(k_{f}\nabla p\right)+\frac{1}{M}\frac{\partial p}{\partial t}=0\;\;\;\;\;\text{on}\;\Omega$$

in which $k_{f}$ denotes the effective permeability coefficient of the porous medium, $k_{f}=k/\mu$. *k* and μ are the intrinsic permeability of the porous medium and the viscosity of the fluid, respectively, and *M* is the Biot modulus:

(5) $$\frac{1}{M}=\frac{\alpha -n_{f}}{K_{s}}+\frac{n_{f}}{K_{f}}$$

with $K_{s}$ and $K_{f}$ the solid and fluid moduli, respectively, and $n_{f}$ the porosity. $\dot{u}$ represents the velocity of the solid and $\dot{□}$ denotes the time derivative:

(6) $$\dot{□}=\frac{\partial □}{\partial t}\;\;\;\dot{u}=\frac{\partial u}{\partial t}$$

As starting point for the derivation of Equation (4), Darcy's law is used:

(7) $$q=-k_{f}\nabla p=n_{f}\left(v-\dot{u}\right)$$

with q the fluid flux and v the velocity of the fluid.

### 2.3 Fluid flow in the fracture

To characterise the fluid flow inside the crack $\Gamma_{c}$, we consider a fully open crack filled with a Newtonian fluid,^24^ see Figure 1A. The crack opening is assumed to be small compared to its length. In a two‐dimensional setting the mass balance for the flow inside the crack reads:

(8) $$\frac{\partial v}{\partial s}+\frac{\partial w}{\partial n}=0$$

with v=v·s and w=v·n being the tangential and normal components of the fluid velocity v in the crack, respectively. s and n are the tangential and normal vectors at the crack $\Gamma_{c}$, see Figure 1A, and (s,n) denotes the local coordinate system along the crack $\Gamma_{c}$. Integrating Equation (8) over the fracture height *h* leads to the difference in the fluid velocity normal to crack faces:

(9) $$w^{+}-w^{-}=-\int_{-h/2}^{h/2}\frac{\partial v}{\partial s}dn$$

where $w^{+}$ and $w^{-}$ are the normal fluid velocities at the interface ${\Gamma_{c}}^{+}$ and ${\Gamma_{c}}^{-}$, respectively. In this contribution, the fracture height *h* is taken as the normal displacement jump in the fracture.

We assume that the tangential fluid velocity *v* inside the crack is much higher than the normal fluid velocity *w*. Since the fracture height *h* is much smaller than its length, we consider a constant fluid pressure $p_{d}$ inside the crack, see Figure 1B. The balance of momentum in the tangential direction is then derived as

(10) $$-\frac{\partial p_{d}}{\partial s}+\frac{\partial \tau }{\partial n}=0$$

with τ being the shear stress, which is derived from $\tau =\mu \frac{\partial v}{\partial n}$. Now, we reformulate Equation (10) as

(11) $$-\frac{\partial p_{d}}{\partial s}+\frac{\partial }{\partial n}\left(\mu \frac{\partial v}{\partial n}\right)=0$$

and the tangential fluid velocity is obtained by solving Equation (11) with no‐slip boundary conditions at n=±h/2:

(12) $$v\left(n\right)=\frac{1}{2\mu }\frac{\partial p_{d}}{\partial s}\left(n^{2}-{\left(\frac{h}{2}\right)}^{2}\right)$$

Substituting Equation (12) into Equation (9) yields

(13) $$w^{+}-w^{-}=\frac{1}{\mu }\frac{\partial }{\partial s}\left(\frac{\partial p_{d}}{\partial s}\frac{h^{3}}{12}\right)$$

To obtain the normal fluid velocity difference in Equation (13), we assign the fluid pressure $p^{+}$ to $\Gamma_{c}^{+}$, $p^{-}$ to $\Gamma_{c}^{-}$ and $p_{d}$ to $\Gamma_{c}^{d}$, respectively. The existence of an independent pressure $p_{d}$ inside the crack allows to model pressurising the crack. The inflow of the fluid through the interface $\Gamma_{c}^{+}$ and $\Gamma_{c}^{-}$ could be different due to the insertion of the interface $\Gamma_{c}^{d}$ and the independent pressure $p_{d}$. In principle, the resistances at $\Gamma_{c}^{+}$ and $\Gamma_{c}^{-}$ can be different, but we assume them to be equal, so that the interface permeability reads $k_{i}$. Analogous to Darcy's law, the normal fluid velocity *w* is determined as^24^:

(14) $$w^{+}=k_{i}\left(p_{d}-p^{+}\right)+\frac{1}{2}\frac{\partial h}{\partial t}\;\;w^{-}=k_{i}\left(p^{-}-p_{d}\right)-\frac{1}{2}\frac{\partial h}{\partial t}$$

Substituting Equation (14) into Equation (13) yields the mass balance equation for the flow within $\Gamma_{c}$

(15) $$k_{i}\left(p_{d}-p^{+}\right)+k_{i}\left(p_{d}-p^{-}\right)+\frac{\partial h}{\partial t}-\frac{\partial }{\partial s}\left(\frac{h^{3}}{12\mu }\frac{\partial p_{d}}{\partial s}\right)=0\;\;\text{on}\;\Gamma_{c}$$

### 2.4 Initial and boundary conditions

The governing equations of the porous medium are completed by the initial and boundary conditions. The Dirichlet boundary conditions read

(16) $$u\left(x\right)=\bar{u}\;\;\;\;\;\;\;\;\;\;\;\text{on}\;\Gamma_{u},\;\;\;\;\;\;\;\;\;\;p\left(x\right)=\bar{p}\;\;\;\;\;\;\;\;\text{on}\;\Gamma_{p}$$

where $\bar{u}$ and $\bar{p}$ denote the prescribed displacement and pressure, respectively. The Neumann boundary conditions are given by

(17) $$\sigma \cdot n=\hat{t}\;\;\;\;\;\;\;\;\;\;\text{on}\;\Gamma_{t},\;\;\;\;\;\;\;\;\;\;q\cdot n=\hat{q}\;\;\;\;\;\text{on}\;\Gamma_{q}$$

in which $\hat{t}$ and $\hat{q}$ represent the prescribed traction and inflow, respectively. For the flow in the fracture, the boundary conditions read

(18) $$p_{d}={\hat{p}}_{d}\;\;\;\;\;\text{on}\;\partial \Gamma_{c}\;\;q_{d}=\hat{Q}\;\;\;\;\;\;\text{on}\;\Gamma_{Q}$$

${\hat{p}}_{d}$ is the pressure imposed on $\partial \Gamma_{c}$. $\hat{Q}$ denotes the inflow imposed on $\Gamma_{Q}$, see Figure 1A.

Finally, the initial conditions give as

(19) $$u\left(x,\;0\right)=u_{0},\;\;\;\;\;\;\;\dot{u}\left(x,\;0\right)={\dot{u}}_{0},\;\;\;\;\;\;\;\;p\left(x,\;0\right)=p_{0}\;\;\;\;\;\text{on}\;\Omega$$

where **u**_0_, ${\dot{u}}_{0}$ and *p*_0_ represent initial displacements, velocities and pressures separately.

### 2.5 Cohesive‐zone model

On the crack faces, we consider traction boundary conditions:

(20) $$\sigma \cdot n=t_{c}=t\left(\left[\left[u\right]\right]\right)-p_{d}n\;\;\;\;\;\;\;\;\text{on}\;\Gamma_{c}$$

with t([[u]]) the tractions due to the influence of the crack interface. In this study, a cohesive‐zone model is applied, where the tractions t([[u]]) are a non‐linear function of the displacement jump across the crack interface $\Gamma_{c}$, which in the local coordinate system (s,n) reads:

(21) $$t_{d}=t_{d}\left(\left[\left[v\right]\right]\right)={\left[t_{s}\;\;\;t_{n}\right]}^{T}$$

with [[v]] being the displacement jump across $\Gamma_{c}$ in the local coordinate system (s,n). The traction vector $t_{d}$ relates to the traction in the global coordinate system via a standard transformation:

(22) $$t=R^{T}t_{d},\;\left[\left[v\right]\right]={\left[\left[\left[v_{s}\right]\right]\;\;\;\left[\left[v_{n}\right]\right]\right]}^{T}=R\left[\left[u\right]\right]=R{\left[\left[\left[u_{x_{1}}\right]\right]\;\;\;\left[\left[u_{x_{2}}\right]\right]\right]}^{T}$$

with R the rotation matrix.^25^

In this study, an exponential traction‐separation law is used, defining the traction in the normal and shear directions as:

(23) $$\left\{\begin{array}{c} t_{n}=t_{u}\exp\left(-\frac{t_{u}}{G_{c}}\kappa \right)\\ t_{s}=d_{\text{int}}\exp\left(h_{s}\kappa \right)\left[\left[v_{s}\right]\right] \end{array}\right.$$

with $t_{u}$ the tensile strength of the material, $G_{c}$ the fracture energy, $d_{\text{int}}$ the initial crack shear stiffness (when κ=0), and $h_{s}=\ln\left(d_{\kappa =1.0}/d_{\text{int}}\right)$. κ is a history parameter, set through a loading function $f=f(\left[\left[v_{n}\right]\right],\left[\left[v_{s}\right]\right],\kappa )$:

(24) $$f=\left[\left[v_{n}\right]\right]\text{or}\left[\left[v_{s}\right]\right]-\kappa ⩽0\;\dot{\kappa }⩾0\;\dot{\kappa }f=0$$

For the unloading (f<0), the tractions are derived from a secant relation. To prevent interpenetration, a penalty stiffness $k_{p}$ is specified in the normal direction.

## 3 WEAK FORM OF THE GOVERNING EQUATIONS

The weak form of balance equations (1), (4) and (15) is obtained through multiplication by the test functions η, ζ and ξ for the solid skeleton, the interstitial pressure and the fluid pressure within the fracture, respectively. Employing the divergence theorem and considering the internal boundaries $\Gamma_{c}^{+}$ and $\Gamma_{c}^{-}$ as well as the conditions at the external boundaries $\Gamma_{u}$, $\Gamma_{t}$, $\Gamma_{p}$ and $\Gamma_{q}$, yields the weak form:

(25a) $$\begin{array}{c} \int_{\Omega }\nabla \eta :\left(\sigma_{s}-\alpha pI\right)d\Omega -\int_{\Gamma_{c}^{+}}\eta^{+}\cdot \left(n^{+}\cdot \sigma^{+}\right)d\Gamma -\int_{\Gamma_{c}^{-}}\eta^{-}\cdot \left(n^{-}\cdot \sigma^{-}\right)d\Gamma =\int_{\Gamma_{t}}\eta \cdot \hat{t}d\Gamma \end{array}$$

(25b) $$\begin{array}{cc} \int_{\Omega }\alpha \zeta \nabla \cdot \dot{u}d\Omega +\int_{\Omega }k_{f}\nabla \zeta \cdot \nabla pd\Omega +\int_{\Omega }\frac{1}{M}\zeta \dot{p}d\Omega & +\int_{\Gamma_{c}^{+}}\zeta^{+}\left(n^{+}\cdot q^{+}\right)d\Gamma \\ +\int_{\Gamma_{c}^{-}}\zeta^{-}\left(n^{-}\cdot q^{-}\right)d\Gamma & =-\int_{\Gamma_{q}}\zeta \hat{q}d\Gamma \end{array}$$

(25c) $$\begin{array}{cc} \int_{\Gamma_{c}}\xi \frac{\partial h}{\partial t}d\Gamma +\int_{\Gamma_{c}}\frac{\partial \xi }{\partial s}\frac{h^{3}}{12\mu }\frac{\partial p_{d}}{\partial s}d\Gamma & -\int_{\Gamma_{c}^{+}}\xi k_{i}\left(p^{+}-p_{d}\right)d\Gamma \\ -\int_{\Gamma_{c}^{-}}\xi k_{i}\left(p^{-}-p_{d}\right)d\Gamma & =\int_{\partial \Gamma_{Q}}\xi \hat{Q}d\Gamma_{Q} \end{array}$$

Considering force equilibrium conditions at crack faces, we have

(26) $$-n^{+}\cdot \sigma^{+}=n^{-}\cdot \sigma^{-}=t\left(\left[\left[u\right]\right]\right)-p_{d}n$$

with $n=n^{-}=-n^{+}$. Reformulating Equation (25a) with the aid of Equation (26) leads to

(27) $$\begin{array}{c} \int_{\Omega }\nabla \eta :\left(\sigma_{s}-\alpha pI\right)d\Omega +\int_{\Gamma_{c}}\left[\left[\eta \right]\right]\cdot \left(t\left(\left[\left[u\right]\right]\right)-p_{d}n\right)d\Gamma =\int_{\Gamma_{t}}\eta \cdot \hat{t}d\Gamma \end{array}$$

with $\left[[\eta ]\right]=\eta^{+}-\eta^{-}$.

The fluid transport across the crack interface $\Gamma_{c}^{+}$ and $\Gamma_{c}^{-}$ is formulated in a Darcy‐like manner^24^:

(28) $$n^{-}\cdot q^{-}=k_{i}\left(p^{-}-p^{d}\right)\;\text{across}\;\Gamma_{c}^{-},\;n^{+}\cdot q^{+}=k_{i}\left(p^{+}-p^{d}\right)\;\text{across}\;\Gamma_{c}^{+}$$

Substituting this equation into the weak form of the mass balance, Equation (25b), results in

(29) $$\begin{array}{cc} \int_{\Omega }\alpha \zeta \nabla \cdot \dot{u}d\Omega +\int_{\Omega }k_{f}\nabla \zeta \cdot \nabla pd\Omega +\int_{\Omega }\frac{1}{M}\zeta \dot{p}d\Omega & -\int_{\Gamma_{c}^{+}}\zeta^{+}k_{i}\left(p^{d}-p^{+}\right)d\Gamma \\ +\int_{\Gamma_{c}^{-}}\zeta^{-}k_{i}\left(p^{-}-p^{d}\right)d\Gamma & =-\int_{\Gamma_{q}}\zeta \hat{q}d\Gamma \end{array}$$

## 4 POWELL‐SABIN FINITE ELEMENT DISCRETISATION

In this section, we will succinctly summarise the finite element discretisation. Powell‐Sabin B‐splines are used to approximate the trial functions in the solution space, and to parameterise the geometry.^26^ Poromechanical interface elements are used to consider the porous effect on the crack faces. The linearised set of equations for the Newton‐Raphson iterative scheme are given as well.

### 4.1 Powell‐Sabin finite elements

**FIGURE 2:**
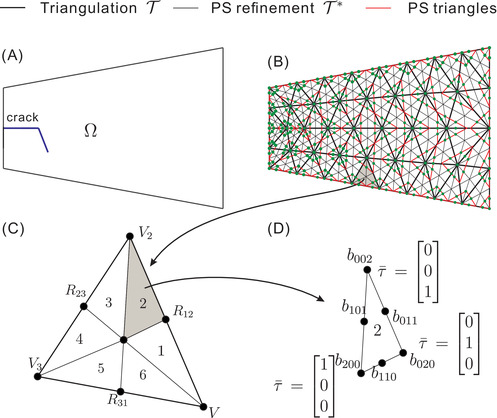
(A) Example of a cracked domain Ω; (B) triangulation T (thick black lines) of the domain, Powell‐Sabin refinement $T^{*}$ (thin black lines) of T, Powell‐Sabin triangles (red) and Powell‐Sabin points (green). In (C) each triangle *e* is subdivided into six mini‐triangles. In (D) each mini‐triangle has a barycentric coordinate system $\bar{\tau }$

Powell‐Sabin B‐splines are employed to discretise Equations (27) and (29). They are defined on triangles, maintaining $C^{1}$‐continuity throughout the whole domain, even at crack tips. They describe the geometry and interpolate the displacement field **u** and the fluid pressure *p* in an isoparametric sense:

(30) $$x=\sum_{k=1}^{N_{v}}\sum_{j=1}^{3}N_{k}^{j}X_{k}^{j}\;u=\sum_{k=1}^{N_{v}}\sum_{j=1}^{3}N_{k}^{j}U_{k}^{j}\;p=\sum_{k=1}^{N_{v}}\sum_{j=1}^{3}N_{k}^{j}p_{k}^{j}$$

where $X_{k}^{j}$ represent the coordinates of Powell‐Sabin triangle corners $Q_{k}^{j}$, see Figure 2B. $U_{k}^{j}$ and $p_{k}^{j}$ denote the degrees of freedom at $Q_{k}^{j}$. The indices j=1,2,3 imply that each vertex *k* has three Powell‐Sabin B‐splines attached, Figure 2B. $N_{v}$ denotes the total number of vertices on the triangulation T.

A cracked domain Ω is considered, cf. Figure 2A. In Figure 2B, it is discretised by a triangulation T, and can be generated by any package for standard triangular elements, such as Gmsh.^27^ On the triangulation T, there are e=1,2,…,E triangles and $N_{v}$ vertices, represented by thick black lines in Figure 2B. Each triangle *e* is split into six (n=1,2,…,6) mini‐triangles, cf. Figure 2B. We perform the Powell‐Sabin refinement $T^{*}$ and get Powell‐Sabin points, green dots in Figure 2B. Then, we define a Powell‐Sabin triangle for each vertex *k*,^25^, ^28^ drawn in red in Figure 2B. Finally, we follow the procedure given in^29^ to define the Powell‐Sabin triangles on the boundary.

The Powell‐Sabin B‐splines $N_{k}^{j}$ over each mini‐triangle in Figure 2C are obtained using the Bézier ordinates $b_{r,s,t}$ ^25^:

(31) $$N_{k}^{j}\left(\bar{\tau }\right)=\sum_{r+s+t=2}b_{r,s,t}B_{r,s,t}^{2}\left(\bar{\tau }\right)\;\text{with}\;B_{r,s,t}^{2}\left(\bar{\tau }\right)=\frac{2!}{r!s!t!}{\bar{\tau }}_{1}^{r}{\bar{\tau }}_{2}^{s}{\bar{\tau }}_{3}^{t}$$

where the subscript *k* denotes the triangle vertex *k* with a coordinate $V_{k}=\left(x_{1}^{k},x_{2}^{k}\right)$. The superscript j=1,2,3 represents the three Powell‐Sabin B‐splines defined on vertex *k*. $\bar{\tau }={\left[\begin{array}{ccc} {\bar{\tau }}_{1} & {\bar{\tau }}_{2} & {\bar{\tau }}_{3} \end{array}\right]}^{T}$ denotes the barycentric coordinate, cf. Figure 2D. $B_{r,s,t}^{2}\left(\bar{\tau }\right)$ represent the Bernstein polynomials. The Bézier ordinates $b_{r,s,t}$ are obtained by considering the properties of Powell‐Sabin B‐splines at each vertex *k*.^26^

We now formulate the Powell‐Sabin B‐splines in a matrix form and implement them in existing finite element codes by Bézier extraction

(32) $$N_{n}^{e}=C_{n}^{e}B$$

where $N_{n}^{e}$ are Powell‐Sabin B‐splines associated with each mini‐triangle in element *e*. B are Bernstein polynomials. $C_{n}^{e}$ is a matrix filled by Bézier ordinates.

Powell‐Sabin B‐splines do not hold the Kronecker‐delta property and are non‐interpolatory at the vertex.^29^ Thus, imposing Dirichlet boundary conditions on $\Gamma_{u}$ and $\Gamma_{p}$ is not as straightforward as for standard finite elements. In this contribution, we will employ Lagrange multipliers to weakly impose essential boundary conditions.^29^

### 4.2 Poromechanical interface elements in the Powell‐Sabin finite element scheme

**FIGURE 3:**
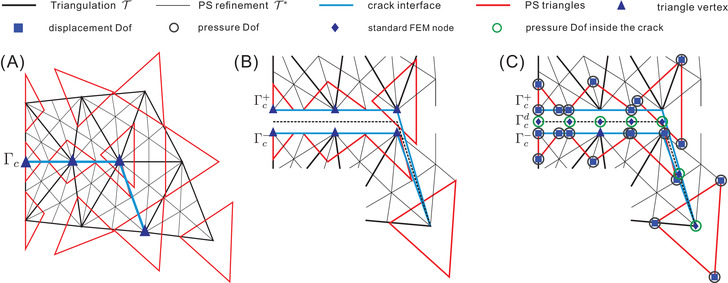
(A) Powell‐Sabin refinement $T^{*}$ (thin black lines), Powell‐Sabin triangles (red) and triangle vertices (blue triangles) along the interface $\Gamma_{c}$; (B) enlargement of the interface $\Gamma_{c}$; (C) zero‐thickness interface elements enriched with pressure degrees of freedom (3PDOF model).

The fluid inside the crack $\Gamma_{c}$ induces a pressure on the crack faces. To include this effect we will apply interface elements enhanced with porosity. Powell‐Sabin B‐splines do not satisfy the Kronecker‐delta property and are non‐interpolatory at the vertex. The augmentation with pressure degrees of freedomis not as standard as in Lagrange basis functions. For the three pressure degrees of freedom (3PDOF) model, three pressure degrees of freedom are added (Figure 3): one on each side of the crack face, and one inside the crack, which allows the discontinuity in the pressure field across the crack $\Gamma_{c}$ and fluid injections along the crack $\Gamma_{c}$.

The 3PDOF model assigns $p^{+}$ to $\Gamma_{c}^{+}$, $p^{-}$ to $\Gamma_{c}^{-}$ and $p_{d}$ to $\Gamma_{c}^{d}$, respectively. To discretise Equation (25c), Powell‐Sabin B‐splines cannot be used because of their definition on triangles, not along a curve. In this study, standard finite elements (FEM) are used to describe the geometry of the interface $\Gamma_{c}^{d}$ and to interpolate the pressure $p_{d}$ inside the interface, see Figure 3C. Here, quadratic Lagrange shape functions are considered.

(33) $$x=\sum_{e=1}^{n_{c}}N_{d}^{e}X_{d}^{e},\;\;p_{d}=\sum_{e=1}^{n_{c}}N_{d}^{e}p_{d}^{e}$$

with $p_{d}^{e}$ nodal degrees of freedom, $N_{d}^{e}$ quadratic Lagrange shape functions, and $X_{d}^{e}$ the coordinates of standard FEM nodes. The FEM nodes are the triangle vertices itself and the middle points between two vertices, see Figure 3C.

The mass conservation Equations (25c) and (29) contain time derivatives, which are discretised using the Backward Euler scheme. Considering the Powell‐Sabin approximation Equation (30) and standard FEM approximation Equation (33), the weak form Equations (25), (29) and (25c) yields:

(34a) $$\begin{array}{cc} \int_{\Omega }B^{T}\sigma_{s}d\Omega -\int_{\Omega }\alpha B^{T}mN_{p}p^{t+\Delta t}d\Omega & +\int_{\Gamma_{c}}H^{T}t\left(\left[\left[u\right]\right]\right)d\Gamma \\ -\int_{\Gamma_{c}}H^{T}nN_{d}p_{d}^{t+\Delta t}d\Gamma & =\int_{\Gamma_{t}}N^{T}\hat{t}d\Gamma \end{array}$$

(34b) $$\begin{array}{cc} & \int_{\Omega }\alpha N_{p}^{T}m^{T}B\left(U^{t}-U^{t+\Delta t}\right)d\Omega +\int_{\Omega }\frac{1}{M}N_{p}^{T}N_{p}\left(p^{t}-p^{t+\Delta t}\right)d\Omega \\ & \;-\Delta t\int_{\Omega }k_{f}B_{p}^{T}B_{p}p^{t+\Delta t}d\Omega -\Delta t\int_{\Gamma_{c}^{+}}k_{i}{\left(N_{p}^{+}\right)}^{T}N_{p}^{+}p^{t+\Delta t}d\Gamma \\ & \;-\Delta t\int_{\Gamma_{c}^{-}}k_{i}{\left(N_{p}^{-}\right)}^{T}N_{p}^{-}p^{t+\Delta t}d\Gamma +\Delta t\int_{\Gamma_{c}^{+}}k_{i}{\left(N_{p}^{+}\right)}^{T}N_{d}p_{d}^{t+\Delta t}d\Gamma \\ & \;+\Delta t\int_{\Gamma_{c}^{-}}k_{i}{\left(N_{p}^{-}\right)}^{T}N_{d}p_{d}^{t+\Delta t}d\Gamma =\Delta t\int_{\Gamma_{p}}N_{p}^{T}\hat{q}d\Gamma \end{array}$$

(34c) $$\begin{array}{cc} & \int_{\Gamma_{c}}N_{d}^{T}n^{T}H\left(U^{t+\Delta t}-U^{t}\right)d\Gamma +\Delta t\int_{\Gamma_{c}}\frac{\partial N_{d}^{T}}{\partial s}\frac{{\left(n^{T}HU^{t+\Delta t}\right)}^{3}}{12\mu }\frac{\partial N_{d}}{\partial s}p_{d}^{t+\Delta t}d\Gamma \\ & \;-\Delta t\int_{\Gamma_{c}^{+}}k_{i}N_{d}^{T}\left(N_{p}^{+}\right)p^{t+\Delta t}d\Gamma -\Delta t\int_{\Gamma_{c}^{-}}k_{i}N_{d}^{T}\left(N_{p}^{-}\right)p^{t+\Delta t}d\Gamma \\ & \;+2\Delta t\int_{\Gamma_{c}}k_{i}N_{d}^{T}N_{d}p_{d}^{t+\Delta t}d\Gamma =\Delta t\int_{\Gamma_{Q}}N_{d}^{T}\hat{Q}\;d\Gamma_{Q} \end{array}$$

where $N_{p}^{+}$ and $N_{p}^{-}$ are shape functions of ***p*** related to interfaces $\Gamma_{c}^{+}$ and $\Gamma_{c}^{-}$, respectively. **N_d_** and $\frac{\partial N_{d}}{\partial s}$ are shape functions and their derivatives related to the pressure degree of freedom ***p***_*d*_ inside the interface $\Gamma_{c}$.

Linearisation of Equation (34) leads to equations for the Newton‐Raphson iterative scheme:

(35) $$\left[\begin{array}{ccc} K_{\text{uu}}^{\Omega }+K_{\text{uu}}^{\Gamma_{c}} & \;K_{\text{up}}^{\Omega } & \;K_{\text{ud}}^{\Gamma_{d}}\\ K_{\text{pu}}^{\Omega } & \;M_{\text{pp}}^{\Omega }+K_{\text{pp}}^{\Omega }+K_{\text{3D}}^{\Gamma_{c}} & \;K_{\text{pd}}^{\Gamma_{d}}\\ K_{\text{du}}^{\Gamma_{d}} & \;K_{\text{dp}}^{\Gamma_{d}} & \;K_{\text{dd}}^{\Gamma_{d}} \end{array}\right]\left[\begin{array}{c} \Delta U\\ \Delta p\\ \Delta p_{d} \end{array}\right]=F_{\text{ext}}-F_{\text{int}}$$

with the tangential stiffness matrices K given in Appendix A. $F_{\text{ext}}$ and $F_{\text{int}}$ are external and internal force vectors, which could be obtained from Equation (34).

## 5 ADAPTIVE ANALYSIS FOR CRACK GROWTH

**FIGURE 4:**
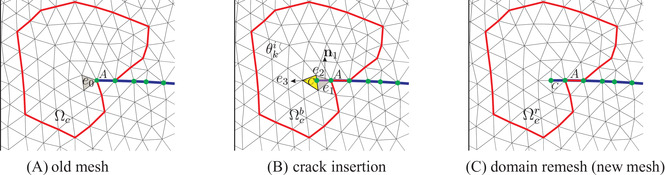
An example of crack insertion in the case of crack propagation. (A) Old mesh before the crack insertion. The blue solid curve denotes the crack interface $\Gamma_{c}$. (B) Crack insertion in the old mesh. Point *A* gives the old crack tip, while point *C* denotes the new crack tip. (C) Domain remesh after the crack insertion. Segment AC represents the new crack interface. $\Omega_{c}$ is the remeshing domain, confined in the red polygon. $\Omega_{c}^{b}$ and $\Omega_{c}^{r}$ are the mesh before and after remeshing $\Omega_{c}$.

Due to the $C^{1}$‐continuity of Powell‐Sabin B‐splines at the crack tip, point *A* in Figure 4A, we can directly assess the fracture criterion at this point. The Rankine criterion has been used here to check crack initiation, comparing the major principal stress σ_1_ with the tensile strength $t_{u}$. If $\sigma_{1}⩾t_{u}$, a crack is inserted through the entire element, *e*_0_ in Figure 4B, in front the crack tip. There is no information about the curvature of the crack segment within the element *e*_0_. Therefore, a straight line is inserted within *e*_0_, shown in Figure 4C. The normal vector $n_{1}$ of the new crack segment, AC in Figure 4 (B, can directly be obtained from the stress tensor at the crack tip. A further improvement of the quality of the crack direction prediction can be obtained by averaging the stress tensor over a finite space around the tip.

After crack insertion element *e*_0_ is divided into two triangles *e*_1_ and *e*_2_, Figure 4B. The triangular element next to the new crack tip, *e*_3_ in Figure 4B, will have four vertices. It is impossible to define Powell‐Sabin B‐splines on this element. Thus, remeshing is needed to remove triangles with four vertices or with a bad aspect ratio. We employ the algorithm proposed in^25^ to remesh the domain, see Figure 4C. In the figure, the original mesh is denoted by $\Omega_{c}^{b}$, while after remeshing the mesh is represented by $\Omega_{c}^{r}$. To determine the domain $\Omega_{c}$ we stand at the element with the newly inserted crack segment, shaded grey in Figure 4A. Then, a radial marching is done until three elements have been crossed in all directions, see Figure 4. The elements along one side of the crack interface are excluded, which avoids updating the field variables along the crack interface.

After remeshing new elements and vertices can be added, and old elements may be moved to ensure elements with a suitable aspect ratio, see Figure 4. Consequently, the mesh is modified and Powell‐Sabin B‐splines are required to be computed on new triangles. Furthermore, due to non‐interpolatory property of Powell‐Sabin B‐splines, the state vector, displacement and interstitial fluid pressure, needs to be mapped from the old mesh $\Omega_{c}^{b}$ onto the new mesh $\Omega_{c}^{r}$ at time step *t* and t+Δt. We take the mapping at time step t+Δt for illustrative purposes. The mapping of the state vector is completed with a least‐square fit subject to certain constraints. We first map the displacement ${}^{t+\Delta t}U$ from $\Omega_{c}^{b}$ to $\Omega_{c}^{r}$:

(36) $$\begin{array}{cc} & \min\;\;\;\;\;\int_{\Omega_{c}}\left\|{}^{t+\Delta t}N_{b}^{u}\;\;{}^{t+\Delta t}U_{b}-{}^{t+\Delta t}N_{r}^{u}\;\;{}^{t+\Delta }U_{r}\right\|d\Omega \;\;\;\;\;\;\\ & \text{subject}\;\text{to:}\;\;W_{\text{int},b}+W_{\text{coh},b}-W_{\text{int},r}-W_{\text{coh},r}=0\;\;\;\;\;\text{on}\;\Omega_{c}^{b}\;\text{and}\;\Omega_{c}^{r}\\ & \;\;\;\;\;\;\;\;\;\;u=\hat{u}\;\;\;\;\;\;\;\;\;\;\;\text{on}\;\Gamma_{\Gamma_{c}}^{u} \end{array}$$

in which the subscript ‘b’ represents the matrix or vector associated with the old mesh $\Omega_{c}^{b}$, while the subscript 'r' related to the new mesh $\Omega_{c}^{r}$. $N^{u}$ denotes the matrix with the shape functions for the displacements. U and u are displacement vectors, and $\Gamma_{c}^{u}$ is the boundary with prescribed displacement. We fix the degree of freedom on the red polygonal boundary and along the crack path of $\Omega_{c}$, see Figure 4C.

In Equation (36), we consider the energy balance as the constraint equation, which matches the energy linked to $\Omega_{c}^{b}$ and $\Omega_{c}^{r}$. The internal work $W_{int}$ and the work $W_{coh}$ related to the cohesive traction on the crack surface are considered due to their direct relation with the displacement u, and given as

(37) $$W_{int}=\int_{\Omega_{c}}\varepsilon :\sigma_{s}d\Omega \;\;\;\;W_{coh}=\int_{\Gamma_{c}}\left[\left[u\right]\right]\cdot \left(t\left[\left[u\right]\right]\right)d\Gamma$$

For the interstitial fluid pressure, the mapping is performed in a similar way as for the displacement, the optimisation problem being

(38) $$\begin{array}{cc} & \min\;\;\;\;\;\int_{\Omega_{c}}\left\|{}^{t+\Delta t}N_{b}^{p}\;\;{}^{t+\Delta t}p_{b}-{}^{t+\Delta t}N_{r}^{p}\;\;{}^{t+\Delta t}p_{r}\right\|d\Omega \;\;\;\;\;\;\\ & \text{subject}\;\text{to:}\;\int_{\Omega_{c}^{b}}k_{f}\nabla p\cdot \nabla pd\Omega -\int_{\Gamma_{c}^{b+}}p^{+}\left(n\cdot q^{+}\right)d\Gamma +\int_{\Gamma_{c}^{b-}}p^{-}\left(n\cdot q^{-}\right)d\Gamma \\ & \;\;\;\;=\int_{\Omega_{c}^{r}}k_{f}\nabla p\cdot \nabla pd\Omega -\int_{\Gamma_{c}^{r+}}p^{+}\left(n\cdot q^{+}\right)d\Gamma +\int_{\Gamma_{c}^{r-}}p^{-}\left(n\cdot q^{-}\right)d\Gamma \\ & \;\;\;\;\;\;\;\;\;\;p=\bar{p}\;\;\;\;\;\;\;\text{on}\;\Gamma_{\Omega_{c}}^{p} \end{array}$$

where $N^{p}$ is the fluid pressure shape function matrix, p is the fluid pressure vector, and $\Gamma_{c}^{p}$ is the boundary with the prescribed fluid pressure. In the constraint equation, we force the mass to be conserved between $\Omega_{c}^{b}$ and $\Omega_{c}^{r}$. Only mass terms directly linked to the fluid pressure *p* are considered. For the fluid pressure $p_{d}$ inside the crack $\Gamma_{c}$, after the crack insertion, we assign zero pressure values to the new crack segment. For the old crack segments, the value does not change. In this study, the MATLAB function *fmincon* is used to find the optimum in Equations (36) and (38). Alternatively, one can use optimisation packages like MOSEK^30^ or ALGLIB,^31^ which may provide a better efficiency for large‐scale problems. In general, the constraint equations from the energy balance and mass conservation reduce the error level of state vector update. A detailed error analysis of the state vector update with a constraint equation for energy conservation under dynamic loading has been carried out in elsewhere.^28^

The computation efficiency in the proposed method is somewhat lower than that in standard finite element analysis. In the evaluation of Equations (36) and (38), we need to find the state vector of Gauss points on the refined mesh from the old mesh.^32^ For each triangular element, there are six mini‐triangles used to carry out the integration. Thus, the number of triangles used in the integration is $N_{e}\times 6$, and the number of Gauss points on the refined mesh is $N_{g}\times N_{e}\times 6$, where $N_{e}$ denotes the total number of triangular elements and $N_{g}$ is the number of Gauss integration points inside each mini‐triangle. In standard FEM the number of Gauss points is $N_{g}\times N_{e}$, which is smaller than that in the proposed method. Thus, the computation time in the proposed method will be increased in comparing to standard FEM.

## 6 NUMERICAL EXAMPLES

Below we will consider two examples. The first example deals with a pre‐fractured specimen, assessing the accuracy of the method. The last example features crack propagation under mixed‐mode loading conditions, demonstrating the ability of the method to analyse the propagation of curved cracks.

### 6.1 Single‐edge notched plate

**FIGURE 5:**
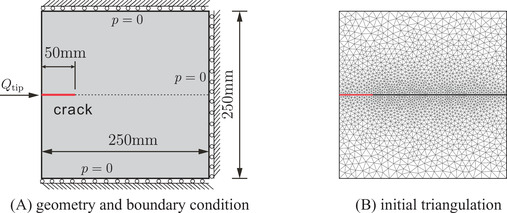
Square plate with an edge crack. (A) Geometry and boundary condition of the problem; (B) initial triangulation of the domain. In the figure, the crack is represented by the red line.

The problem consists of a square plate with dimensions 250mm×250mm, with a horizontal crack through the centre of the plate, shown in Figure 5. The first 50mm of the crack is pre‐fractured, and an inflow of $Q_{tip}=50\;{\text{mm}}^{2}/s$ is imposed on the left end of the crack. The pressure is zero at the top, bottom and right boundaries. The displacement in the horizontal direction is constrained at the right boundary, while the displacement in the vertical direction is fixed at the top and bottom boundaries, see Figure 5A. The following material properties are employed in the simulation: Young's modulus $25.85\times 10^{3}\;\text{MPa}$, Poisson's ratio ν=0.18, porosity $n_{f}=0.2$, intrinsic permeability $k=2.78\times 10^{-16}\;m^{2}$, viscosity $\mu =10^{-9}\;\text{MPa}\;s$, Biot coefficient α=1, bulk modulus of the solid $K_{s}=13.46\times 10^{3}\;\text{MPa}$ and the fluid modulus $K_{f}=200\;\text{MPa}$. The cohesive zone model in Equation (23) is used with the tensile strength $t_{u}=2.7\;\text{MPa}$ and fracture energy $G_{c}=0.095\;\text{N/mm}$. Only mode I fracture is considered, that is, $d_{\text{int}}=0$ in Equation (23). To avoid interpenetration, a penalty stiffness $k_{p}=10^{10}\;\text{MPa/mm}$ is specified in the normal direction of the crack. The interface permeability, $k_{i}$, is set as the effective permeability coefficient of the porous medium $k_{f}$. The plate has been discretised by the triangulation presented in Figure 5B. A constant time step size Δt=0.01s is used in the simulation.

**FIGURE 6:**
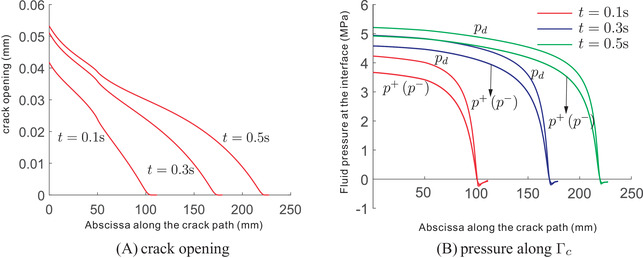
Crack opening (A) and pressure (B) along the crack interface $\Gamma_{c}$.

**FIGURE 7:**
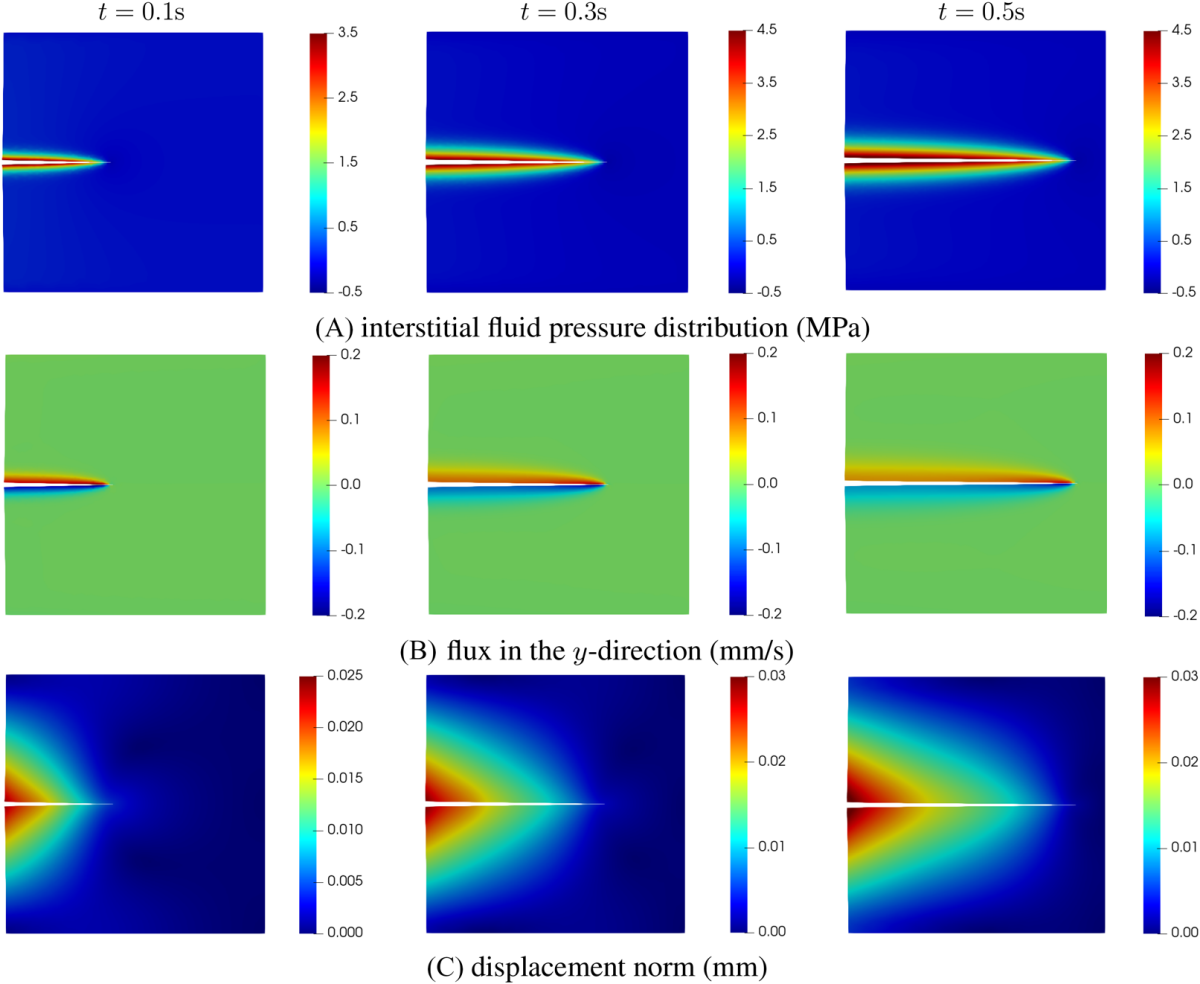
Interstitial fluid pressure, flux in the *y*‐direction ($q_{y}=-k_{f}\partial p/\partial y$) and displacement norm. Each column presents the results at time step *t*. The displacements have been amplified by a factor 100.

Figure 6A shows the crack opening along the crack interface $\Gamma_{c}$ for different time steps. Obviously, the crack opening increases as the time evolves. This evolution is also evident from the pressure along the crack interface, see Figure 6B. In the figure, the pressure inside the crack is higher than the pressure on either side of the interface. The results match well with those in ref.,^8^ validating the proposed method. Indeed, in ref.^8^ the set‐up of the problem is similar to the KGD problem.^33^, ^34^ Figure 7 presents the contour plot of the interstitial fluid pressure, the flux in the *y*‐direction and the displacement norm. As shown in the Figure 7A, almost the entire crack interface $\Gamma_{c}$ is pressurised due to the flow injection at the left edge, except the small part around the crack tip. The pressurisation along the crack interface can also be observed from the flux profile in the *y*‐direction, that is Figure 7B. Due to the symmetric setup of the problem, the displacement profile shows a symmetric distribution along the middle plane of the plate, see Figure 7B.

### 6.2 Arbitrary propagation: A plate with two propagating cracks

**FIGURE 8:**
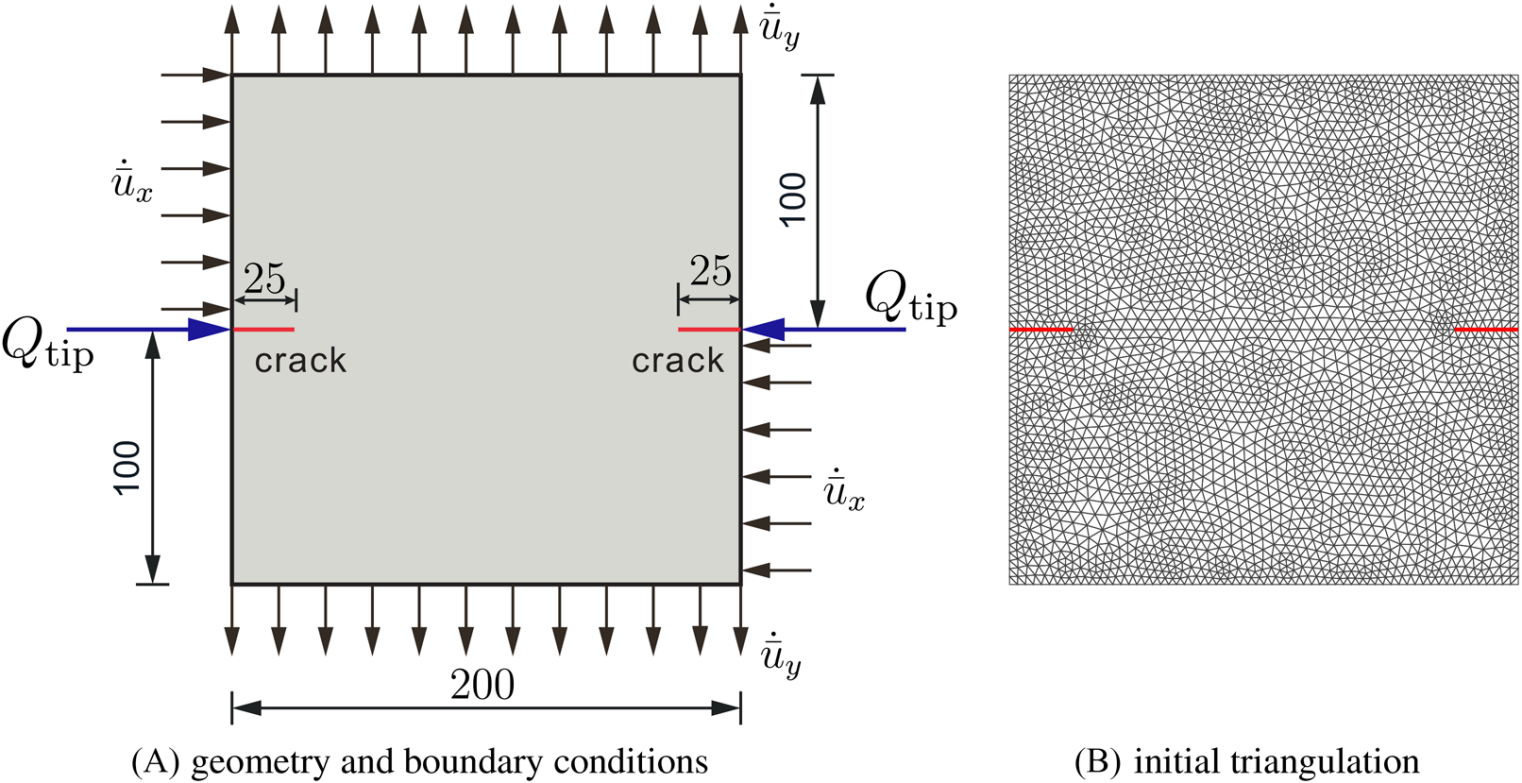
A plate with two propagating cracks.

We next consider the specimen of Figure 8 to demonstrate the ability of the proposed method to properly analyse mixed‐mode crack problems. The specimen has a thickness of 50 mm and has two initial horizontal notches. Figure 8 (A shows the geometry and the boundary conditions. In the analysis, the specimen is subjected to a prescribed horizontal velocity ${\dot{\bar{u}}}_{x}$ and a vertical velocity ${\dot{\bar{u}}}_{y}$. Fluid is injected at the inlet of the initial notches at a constant rate $Q_{tip}$. The time increment is set as Δt=0.005s.

The following material parameters are used in the analysis: Young's modulus E=30GPa, Poisson's ratio ν=0.2, Biot coefficient α=1, porosity $n_{f}=0.2$, intrinsic permeability $k=2.78\times 10^{-16}\;m^{2}$, solid bulk modulus $K_{s}=13.46\times 10^{3}\;\text{MPa}$, fluid bulk modulus $K_{f}=200\;\text{MPa}$, fluid viscosity $\mu =10^{-9}\;\text{MPa}\;s$. The exponential decohesion relation in Equation (23) is employed to describe the fracturing process with a tensile strength $t_{u}=3.0\;\text{MPa}$ and a fracture energy $G_{c}=0.11\;\text{N/mm}$. Mode‐II crack behaviour is considered: $d_{\text{int}}=10\;\text{N/mm}$ and $h_{s}=0$ in Equation (23).^35^ Plane‐stress conditions are assumed and the loading condition is set up as:

Step 1 The displacement in the *x*‐direction is constrained at the upper left and bottom right edges. The top and bottom edges are fixed in the *y*‐direction. Correspondingly, we have the value of the velocities: ${\dot{\bar{u}}}_{x}=0\;\text{mm/s}$ and ${\dot{\bar{u}}}_{y}=0\;\text{mm/s}$ at this stage. Fluids are injected at the inlet of pre‐fractured cracks at a value $Q_{tip}=50\;{\text{mm}}^{2}/s$. The total loading time is T=0.05s.
Step 2 The specimen is then subjected to a prescribed horizontal and vertical velocity ${\dot{\bar{u}}}_{x}={\dot{\bar{u}}}_{y}=2\times 10^{-2}\;\text{mm/s}$, see Figure 8. A displacement control is employed to apply the velocity ${\dot{\bar{u}}}_{x}$ and ${\dot{\bar{u}}}_{y}$ in the simulation, $\Delta u_{x}=\Delta u_{y}={\dot{\bar{u}}}_{x}\times \Delta t={\dot{\bar{u}}}_{y}\times \Delta t=1.0\times 10^{-4}\;\text{mm}$. The displacement is imposed by Lagrange multiplier method in.^29^ At this stage, fluid injects are terminated.

**FIGURE 9:**
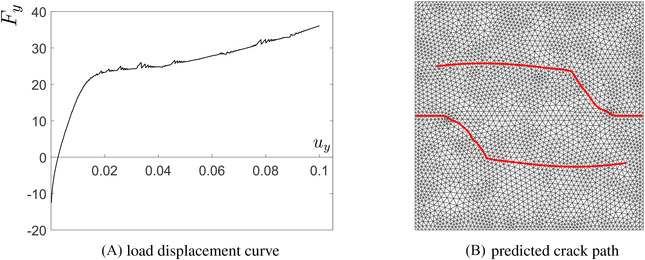
Load (kN)‐displacement (mm) response and predicted crack path.

**FIGURE 10:**
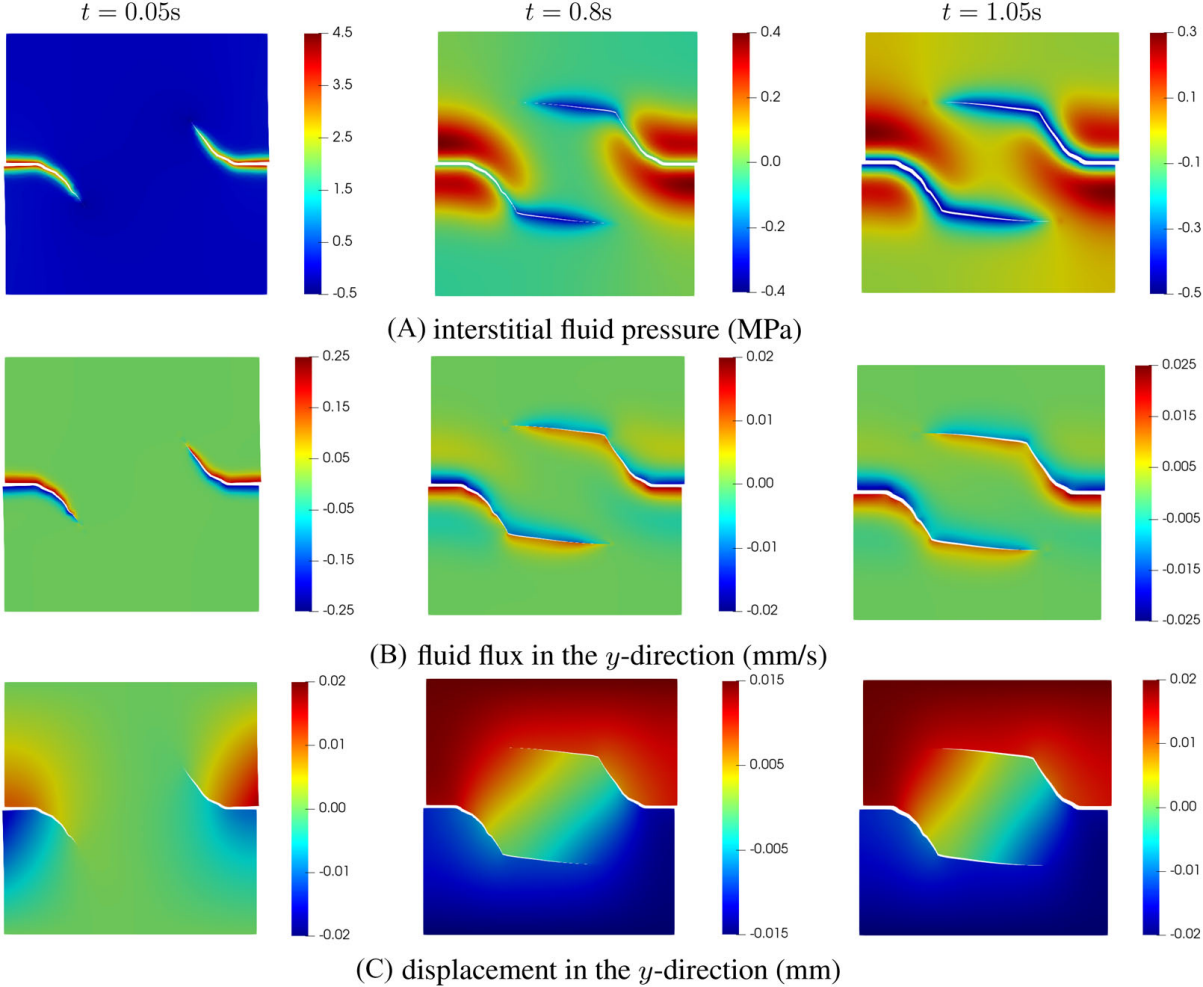
Interstitial fluid pressure, the flux and displacement in the *y*‐direction at different time steps. The displacements have been amplified by a factor 100.

The load‐displacement diagram in Figure 9A presents the relation between the vertical resultant force $F_{y}$ and the vertical displacement $u_{y}$ on the top edge at loading Step 2. In the process of crack propagation, the fluid pressure inside the crack and on the crack interface will gradually impose a tensile stress (negative fluid pressure) on the crack faces, see Figure 10A‐middle and 10A‐right. In the course of time nearly the entire crack interface will come in tension, as illustrated in Figure 10A‐right, which induces the increase of the force on the top panel. The prediction of the crack path is presented in Figure 9B. The curved crack path shows the refinement ability of the Powell‐Sabin B‐splines. There is a kink on the crack path, due to the change of loading conditions from Step 1 to Step 2. In Figure 10, the profiles of the interstitial fluid pressure, the flux and the displacement are illustrated. At the loading Step 1, the crack interface is pressured due to the fluid injection $Q_{tip}$, Figure 10A‐left. The pressurisation can also be observed in the flux plot, Figure 10B‐left. After terminating fluid injection at loading Step 2, the fluid will gradually flow back into the fracture, Figure 10A‐middle and 10A‐right. The flux profile also presents this flowing back, see Figure 10B‐middle and 10B‐right. The displacement in the *y*‐direction is increases as time evolves due to the monotonic loading conditions, Figure 10C.

## 7 CONCLUSIONS

Powell‐Sabin B‐splines have been used in the analysis of hydraulic fracturing. Cohesive interface elements are employed to model the interface behaviour of the solid part, while a three‐pressure degree of freedom model describes the fluid flow inside the fracture.

Powell‐Sabin B‐splines are $C^{1}$‐continuous throughout the domain, even at crack tips. Such higher‐order continuity remedies the inaccurate stress issue in employing Lagrange basis functions. Due to the definition of Powell‐Sabin B‐splines on triangles, crack insertions and crack path tracking are directly performed in the physical domain, circumventing the initial mesh alignment issue in the isogeometric analysis. After inserting new crack segments, remeshing is required to avoid elements with unsuitable aspect ratios, which necessitates a mappling of the state vector from the old onto the new mesh. A novel least‐square fit methodology is introduced in combination with constraint equations from the energy balance and mass conservation.

Numerical examples show that the refinement ability of the Powell‐Sabin B‐splines is very suitable for the analysis of hydraulic fracturing. When fluids are injected into the fracture, the crack interface will be pressured, rendering an increase of the crack opening and forcing the crack propagate. Supposing that no fluids are injected into the fracture, under tensile loading conditions, the existence of fractures will induce tensile stresses on the crack interface, preventing crack openings. The fluid then flows from the porous medium into the fracture.

The extension of Powell‐Sabin B‐splines to three‐dimensional problems is non‐trivial due to certain constraints with neighboring tetrahedrons.^36^, ^37^ Alternatively, one can construct prisms as a tensor product of two‐dimensional Powell‐Sabin B‐splines and Non‐Uniform Rational Basis splines (NURBS) in the third dimension.

## Data Availability

Data sharing not applicable to this article as no datasets were generated or analysed during the current study.

## APPENDIX A TANGENTIAL STIFFNESS MATRIX

The tangential stiffness matrices in Equation (35) are given as

(A.1)
$$\begin{array}{cc} & K_{\text{uu}}^{\Omega }=\int_{\Omega }B^{T}DBd\Omega \;\;\;K_{\text{uu}}^{\Gamma_{c}}=\int_{\Gamma_{c}}H^{T}R^{T}T_{d}RHd\Gamma \\ & K_{\text{up}}^{\Omega }=-\int_{\Omega }\alpha B^{T}mN_{p}d\Omega \;\;K_{\text{pu}}^{\Omega }={\left(K_{\text{up}}^{\Omega }\right)}^{T}\\ & M_{\text{pp}}^{\Omega }=-\int_{\Omega }\frac{1}{M}N_{p}^{T}N_{p}d\Omega \;\;K_{\text{pp}}^{\Omega }=-\Delta t\int_{\Omega }k_{f}B_{p}^{T}B_{p}d\Omega \\ & K_{\text{3D}}^{\Gamma_{c}}=-\Delta t\int_{\Gamma_{c}^{+}}k_{i}{\left(N_{p}^{+}\right)}^{T}N_{p}^{+}d\Gamma -\Delta t\int_{\Gamma_{c}^{-}}k_{i}{\left(N_{p}^{-}\right)}^{T}N_{p}^{-}d\Gamma \\ & K_{\text{ud}}^{\Gamma_{d}}=-\int_{\Gamma_{c}}H^{T}nN_{d}d\Gamma \\ & K_{\text{pd}}^{\Gamma_{d}}=\Delta t\int_{\Gamma_{c}^{+}}k_{i}{\left(N_{p}^{+}\right)}^{T}N_{d}d\Gamma +\Delta t\int_{\Gamma_{c}^{-}}k_{i}{\left(N_{p}^{-}\right)}^{T}N_{d}d\Gamma \\ & K_{\text{du}}^{\Gamma_{d}}=\int_{\Gamma_{c}}N_{d}^{T}n^{T}Hd\Gamma +\Delta t\int_{\Gamma_{c}}\frac{\partial N_{d}^{T}}{\partial s}\frac{{\left(n^{T}HU^{t+\Delta t}\right)}^{2}}{4\mu }n^{T}H\left(\frac{\partial N_{d}}{\partial s}p_{d}^{t+\Delta t}\right)d\Gamma \\ & K_{\text{dp}}^{\Gamma_{d}}=-\Delta t\int_{\Gamma_{c}^{+}}k_{i}N_{d}^{T}\left(N_{p}^{+}\right)d\Gamma -\Delta t\int_{\Gamma_{c}^{-}}k_{i}N_{d}^{T}\left(N_{p}^{-}\right)d\Gamma \\ & K_{\text{dd}}^{\Gamma_{d}}=2\Delta t\int_{\Gamma_{c}}k_{i}N_{d}^{T}N_{d}d\Gamma +\Delta t\int_{\Gamma_{c}}\frac{\partial N_{d}^{T}}{\partial s}\frac{{\left(n^{T}HU^{t+\Delta t}\right)}^{3}}{12\mu }\frac{\partial N_{d}}{\partial s}d\Gamma \end{array}$$

with R the rotation matrix,^25^ $T_{d}$ the tangent stiffness of traction‐opening law at the interface $\Gamma_{c}$.^25^
